# Potential limitations of novel efforts to accelerate and streamline urgent inpatient critical limb revascularization in current vascular surgery practice

**DOI:** 10.1016/j.jvscit.2023.101300

**Published:** 2023-08-22

**Authors:** Joseph Patrick Hart

**Affiliations:** Division of Vascular and Endovascular Surgery, Department of Surgery, Medical College of Wisconsin, Milwaukee, WI

Speirs et al^1^ are due congratulations for their efforts to accelerate and prioritize urgent limb revascularization. Importantly, they raise the concept of formal geriatric review before revascularization, which is often omitted in the United States.^2^ They also maintained a relatively stable complication rate, length of stay, and hospital mortality within the data set they reported in their analysis. At a time when U.S. government reimbursement is effectively decreasing because of payment cuts, the incentives offered by the National Health Service are also both remarkable and noteworthy.

In the United States, we must continue to push our specialty to achieve similar gains. Through the valuation task force, the Society for Vascular Surgery has increased the focus on the value of the work provided by vascular surgeons.^3^, ^4^, ^5^ Undoubtedly, we should lead the charge on these sorts of critical improvements. If we do not, it will be done by governmental bodies, interest groups, watchdog agencies, and/or other specialties. We must retain leadership in this vital arena, and the study by Speirs et al^1^ provides one model to learn from for those pursuing this work in the United States.

Such accelerated protocols can be difficult to enact with similar results everywhere. It is uncertain whether a significant length of stay increase would be associated with such an initiative in U.S. hospital systems.^6^^,^^7^ Most centers perform truly emergent bypasses for trauma or acute ischemia well. With current staffing shortages and operating room access challenges, the broad application of similar efforts could lead to increases in out-of-hour surgeries with significant potential downstream negative results.^1^ Vascular surgeons operate at night or on weekends when necessary. Nevertheless, this takes a toll on teams, leads to more intraoperative staff changes, and has documented challenges regarding increased complications and failure to rescue. Current patient satisfaction efforts in the United States would not allow recurring cancellations and the movement of elective cases to accommodate such urgent cases. Placing such extra, relatively acute volumes at a reasonable time of day from such efforts for a largescale system-wide attempt would be even more challenging. Nevertheless, we must find methods to speed revascularization in a subset of our patients. Defining that subset properly and how to best move forward without unduly taxing the existing system, our teams, and, ultimately, the patients and their outcomes are the issues we must untangle.

Moreover, an open vs endovascular approach is a whole other hornet's nest of perennial questions that have recently seen precious additional data arise.^8^, ^9^, ^10^, ^11^, ^12^ We likely should be doing more open revascularizations, but additional questions remain here as well.^11^^,^^12^ More operative bypasses with potentially fewer available resources than typically provided during daytime revascularizations enhance these concerns.

Vascular surgeons are best suited to address this problem.^3^ It is an urgent matter for our patients and the healthcare system that we do so thoughtfully, rapidly, and with the integrity we seek to bring to all we do as a specialty. This report by Speirs et al^1^ is a valuable addition to the knowledge base that will drive such efforts.
